# Effects of sesame dehulling on physicochemical and sensorial properties of its oil

**DOI:** 10.1002/fsn3.3608

**Published:** 2023-08-29

**Authors:** Maryam Amini, Mohammad Taghi Golmakani, Azam Abbasi, Marzieh Nader

**Affiliations:** ^1^ Shiraz University of Medical Sciences Shiraz Iran; ^2^ School of Agriculture Shiraz University Shiraz Iran; ^3^ Nutrition Research Center, Department of Food Hygiene and Quality Control, School of Nutrition and Food Sciences Shiraz University of Medical Sciences Shiraz Iran

**Keywords:** aflatoxin, antinutritional, hulled, sesame oil, un‐hulled

## Abstract

At present, sesame oil is extracted from un‐hulled white sesame seeds by using cold press lubrication machines in local stores in Iran. This study aimed to evaluate the physicochemical properties and safety parameters of the hulled and un‐hulled white sesame oils. The fatty acid composition, antioxidant activity, oxalates content, total phenolic content, carotenoid content, acid value, peroxide value, p‐anisidine value, value total oxidation value (TOTOX), aflatoxins and pesticides residue, smoke point, color, relative density, and refractive index of oil sample were examined immediately after extracting the oil. The peroxide, p‐anisidine, and TOTOX value of the hulled and un‐hulled sesame oil samples were also examined periodically. After 7 months, the quality parameters were high and the oil samples were not consumable. Linoleic and oleic acids were the predominant fatty acids in the hulled and un‐hulled sesame oils. The results of this study showed that the oil extracted from raw un‐hulled sesame had a lower initial quality than hulled sesame oil and was oxidized more rapidly than it during the storage period. Virgin oils contained impurities acting like prooxidants and reduced their stability and shelf life. In addition, the un‐hulled sesame oil contained higher amounts of antinutrient compounds (e.g., oxalate and pesticide residues) than the hulled sesame oil. Aflatoxin was not detected in our oil samples.

## Graphical abstract

The results of this study showed that the oil extracted from raw un‐hulled sesame had a lower initial quality than hulled sesame oil and oxidized more rapidly during the storage period.

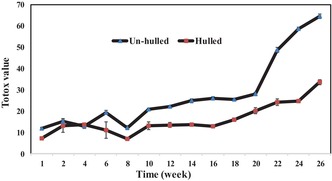

## 1 INTRODUCTION

Oilseeds have an important role in the nutrition and diet of people in terms of energy supply, essential fatty acids, and plant proteins. Sesame (*Sesamum indicum* L.) is the oldest oilseed known by humans (Ashjaei & Asadolahi, 2018). The chemical composition of sesame is 50%–60% oil, 18%–25% protein, 13.5% carbohydrate, and 5% ash. Sesame oil contains a large amount of polyunsaturated fatty acids (PUFAs) and natural antioxidants such as sesamol and sesamolin, which can increase oxidation resistance and stimulate anticoagulant, antihypertensive, anti‐inflammatory, and antidepressant activities (Kim et al., 2014; Langyan et al., 2022; Papadakis et al., 2006).

Sesame hull also contains some antinutritional elements such as oxalic acid, allergen, or antioxidant composition (Hakme et al., 2018; Moazzami et al., 2006; Papadakis et al., 2006; Pastorello et al., 2001; Shahidi, 2006). According to Shahidi (2006), the oxidative stability of oil extracted from the un‐hulled sesame was higher than that of the hulled sesame. However, different processes (e.g., agricultural practices and environmental pollution) could increase the toxic residues in the oil. Several studies have shown the presence of pesticide residues, heavy metals, and mycotoxins in the edible oils and fats (Niu et al., 2021; Xia et al., 2021).

Pesticides and mycotoxins have adverse effects on the environment and produce side effects such as endocrine disorders and damaging liver function. Carcinogenic effect of these compounds resulted from their chemical stability and high lipophilic properties causing bioaccumulation and increased level of human and animal exposure (Hakme et al., 2018; Li et al., 2014).

Some of these toxicant agents are present on the surface of seed or fruit and cannot penetrate into seed or fruit. Therefore, dehulling reduces the amount of residual pesticides and antinutrient compounds and as well as improves the nutritional value and color of the sesame products (Hakme et al., 2018; Hossini et al., 2019; Makinde & Akinoso, 2013). Cui et al. (2022) investigated organochlorine pesticides and other pesticides in peanut oil obtained from shelled and unshelled peanuts and found that the concentration of pesticide in the oil obtained from unshelled peanuts was higher than that in shelled peanuts. Kheirati Rounizi et al. (2021) showed that Ardeh oil extracted using traditional extraction method had a lower quality than the oil extracted using industrial method in terms of chemical characteristics (i.e., higher peroxide, anisidine, and acid values [AVs]).

To our knowledge, no prior study had investigated the effect of hulling process on the safety and quality of sesame oil. Therefore, this study aimed to examine the physicochemical quality of virgin hulled and un‐hulled sesame oils as well as to evaluate the oxidative stability of hulled and un‐hulled sesame oil samples.

## 2 MATERIALS AND METHODS

### 2.1 Preparation of hulled and un‐hulled sesame oils

White sesame was purchased from a grocery store in Shiraz, Iran. Half of the seeds were winnowed, hulled, soaked, and slightly roasted in a peeling workshop, and the remaining half was left un‐hulled. Seeds were roasted at 90°C, 6% humidity. Then, the oils of the samples were extracted by adopting the cold pressing method and using Cold Press Machine (BD 45 Eco Cold Press Machine). Oil extraction was obtained at a pressure of 12 MPa, a temperature of 30 ± 1°C, and a sample value of 10 kg/h (Ghiasi et al., 2022). After extracting the oil and settling the residues, the oils were packaged in 20‐mL dark‐brown bottles and stored in refrigerator (45°C) for 7 months.

### 2.2 Physicochemical properties of hulled and un‐hulled sesame oil samples immediately after extracting the oil

#### 2.2.1 Fatty acid profile

The fatty acid profiles of the hulled and un‐hulled sesame oils were determined according to the method of Alavi and Golmakani (2017) and were analyzed by GC/FID (2017). The temperatures of injector and detector were at 250 and 300°C, respectively. Nitrogen was used as a carrier gas. The results were expressed as the percentage for each detected fatty acid in the oil (Alavi & Golmakani, 2017).

#### 2.2.2 Acid value

To determine the AVs of hulled and un‐hulled sesame oil, 5 g of the oil sample was homogenized with 10 mL of ethanol. Then, phenolphthalein reagent was added to the solution. The resulting solution was titrated with potassium hydroxide (0.1 N) until its color changed to pink. AV was calculated according to Equation (1) (AOCS Official Methods, Cd, d3‐63, 1997):

(1) $$\mathrm{AV}\;\left(g\frac{\mathrm{KOH}}{\mathrm{kg}}\right)=\frac{N\times v\times 56.1}{w}$$

where *N* is the normality of potassium hydroxide, *V* is the volume of sodium hydroxide solution used by the sample, and *W* is the weight of the oil sample.

#### 2.2.3 Peroxide value

Peroxide value (PV) was measured by implementing the iodometric procedure. To this end, 5 g of the oils was initially dissolved in 30 mL of acetic acid: chloroform solution (ratio 3:2). Then, 0.5 mL of saturated potassium iodide was added to the resulting solution. After 1 min, 5 mL of the distilled water and starch reagent (1%) were added, and the resulting solution was titrated with potassium hydroxide (0.1 N). The control solution was prepared by taking the above‐mentioned steps without adding the oil sample. Finally, the PV was calculated using the following Equation (2) (Alavi & Golmakani, 2017):

(2) $$\mathrm{PV}\;\left(m\frac{\mathrm{Eq}}{\mathrm{kg}}\right)=\frac{\left(\left(V_{s}\;–V_{b}\;\right)\times N\times 1000\right)}{W}$$

where *V*_S_ is the volume of sodium hydroxide solution used by the oil sample, *V*_b_ is the volume of sodium hydroxide solution used by the control sample, *W* is the weight of the oil sample, and *N* is the normality of the used potassium hydroxide.

#### 2.2.4 Para‐anisidine value

Para‐anisidine value (p‐AnV) was determined using isooctane solvent and anisidine reagent (0.25% (w/v) methoxy aniline in acetic acid). Absorption at 350 nm was measured by a Dynamica double‐beam ultraviolet–visible spectrophotometer (Halo DB‐20R), and the anisidine value was calculated using the following Equation (3) (AOCS Official Methods, Cd, 18‐90, 1997):

(3) $$p‐A.V\;\left(\frac{\mathrm{mg}}{\mathrm{kg}}\right)=\frac{25\;\left(\;1.2\;A_{b}–A_{a}\right)}{W}$$

where *A*_b_ is the absorption of the oil sample at a wavelength of 350 nm, *A*_a_ is the absorption of the control sample at a wavelength of 350 nm, and *W* is the weight of the oil sample.

#### 2.2.5 Total oxidation (TOTOX) value

TOTOX value was calculated using peroxide/p‐AnVs and employing the following Equation (4) (Alavi & Golmakani, 2017):

(4) $$\text{TOTOX}=2\;\left(\mathrm{PV}\right)+p-A.V$$

#### 2.2.6 Antioxidant capacity

Antioxidant activity of the hulled and un‐hulled oils was determined using the DPPH (2,2‐diphenylpicrylhydrazyl) assay. Briefly, DPPH solution was prepared using the ethyl acetate solvent at a concentration of 0.4 mM. In a test tube, 390 μL of DPPH solution was added to the solution containing 10 μg of oil and 100 μL of ethyl acetate and stirred vigorously for 10 s. The resulting solution was kept in the dark at 22°C for 30 min. Then, the adsorption of the prepared oil sample in ethyl acetate solvent was read using Dynamica double‐beam ultraviolet–visible spectrophotometer (Halo DB‐20R) at a wavelength of 517 nm. A control solution was performed by replacing the oil with pure methanol. The percentage inhibition of the DPPH radical was calculated using the following Equation (5) (Zareie et al., 2021):

(5) $$\%\text{Inhibition of DPPH radical}=\frac{A\;\left(\text{control}\right)–A\;\left(\text{sample}\right)}{A\;\left(\text{control}\right)}\times 100$$

where *A* (sample) is the absorption of oil sample at 517 nm in ethyl acetate and *A* (control) is the absorption of control sample at 517 nm in ethyl acetate.

#### 2.2.7 Oxalates

To determine the oxalate content, 1 g of the oil sample was stirred continuously with 7 mL of 3 M sulfuric acid for 1 h. Then, 25 mL of the sample was titrated with potassium permanganate (0.1 N) solution and passed through Whatman grade 1 filter paper until its color changed to pink (Makinde & Akinoso, 2014). The oxalate concentration of the sample was determined based on the following equation: 1 mL of 0.1 potassium permanganate is equal to 6.303 mg of oxalate.

#### 2.2.8 Total phenolic content

To measure the total phenolic content (TPC), 2.5 g of the oil sample was stirred with 5 mL of hexane and 5 mL of 60% methanol solution for 2 min. Then, the solution was centrifuged for 10 min at 3500 rpm. Next, 0.2 mL of the methanolic extract was diluted with water to a volume of 5 mL, and then 0.5 mL of Folin–Ciocalteu reagent was added to it. After 3 min and adding 1 mL of 35% sodium carbonate solution, the solution was diluted with water to a volume of 10 mL. Then, the absorption rate of the solution was read at 725 nm. Finally, the TPC was evaluated in terms of milligram equivalent of gallic acid per gram of the sample weight by using the standard gallic acid calibration curve (0.05–0.5 mg/mL) (Alavi & Golmakani, 2017).

#### 2.2.9 Carotenoids

The number of carotenoids was determined by adding 25 mL of cyclohexane to 7.5 g of the oil. Then, the absorption rate of the solution was read by spectrophotometer at a wavelength of 470 nm. The carotenoids were calculated using the following Equation (6) (Borchani et al., 2010):

(6) $$\text{Carotenoid content}\;\left(\mathrm{mg}\right)=\frac{A_{470}\times 2.5\times 10^{5}}{E_{0}\times 7.5}$$

where $A_{470}$is the maximum absorption at 470 nm and $E_{0}$is the specific coefficient (2000).

#### 2.2.10 Smoke point

To determine the smoke point, 10 g of the oil sample was heated until the solution started to smoke. Then, a special digital thermometer was placed in the oil container to record the smoke point (AOCS Official Method, Cc 9a‐48, 1997).

#### 2.2.11 Color

Spectrophotometric method is a standard method for measuring the color of oils. The oil sample was diluted with methanol (1:10 ratio), and their absorption was measured at 460, 550, 620, and 670 nm using the Dynamica double‐beam ultraviolet–visible spectrophotometer (Halo DB‐20R). Finally, the color was calculated in terms of the Lovibond red unit using the following Equation (7) (AOCS Official Method Cc 13e‐92, 2017):

(7) $$\text{Color}\;\left(\text{Lovibond}\;\mathrm{red}\right)=1.29A_{460}+69.7A_{550}+41.2A_{620}-56.4A_{670}$$

#### 2.2.12 Relative density

The densitometer was dried and carefully weighed. Then, it was filled with boiled distilled water (without air) and placed in water at 20°C for 30 min. After ensuring the heat balance, it was taken out of the water, dried, and weighed again. To facilitate the drying process, the densitometer was washed several times with alcohol and ether, and was filled with liquid oil at 20°C so that the capillary tube was completely filled with oil. The densitometer was then placed in a water bath at 20°C for 30 min. After the completion of the heat exchange process, it was dried and weighed again and the calculations were performed using Equation (8) (AOCS Official Method, Cc 10c‐95, 2020):

(8) $$\text{Relative density}=\frac{\text{Weight of oil}\;\left(g\right)}{\text{Weight of water}\;\left(g\right)}$$

#### 2.2.13 Refractive index

Refractometer *Atago* RX5000 was used to determine the refractive index. Some drops of homogenized liquid sesame oil with a temperature of 25°C were placed on the prism. The lines of sight were adjusted and the refractive index was read from the graduated plate after reaching the thermal equilibrium within a few minutes (AOCS Official Method, Cc 7‐25, 2017).

#### 2.2.14 Pesticides

Triazophos, bifenthrin, pyriproxyfen, cyhalothrin‐lambda I, cyhalothrin‐lambda II, cyhalothrin‐lambda total, and chlorpyrifos were measured according to British Standard method for hulled and un‐hulled sesame oil samples (BS EN 15662, 2008).

#### 2.2.15 Aflatoxins

The levels of aflatoxins B1, B2, G1, and G2 as well as the total aflatoxins in oil samples were measured according to the method developed by Zhao et al. (2017).

#### 2.2.16 Sensory evaluation

The sensory properties of hulled and un‐hulled sesame oils were compared with those of the refined sample. The test was carried out using a 12‐trained panelist performing a Due‐Trio test. To this end, some evaluators were asked to evaluate the hulled, un‐hulled, and refined oil samples for smell, color, taste, and overall acceptance. The best sample among the given indicators was allocated a score of 3, and the worst sample was given a score of 1. Then, the total scores obtained by each evaluator for each feature were calculated separately. Finally, the tables designed by Kramer test were used for statistical analysis (Amerine et al., 1965).

#### 2.2.17 Oxidative stability during the storage period

The oxidative stability of the hulled and un‐hulled sesame oils was checked every 2 weeks during the 7‐month storage period by examining the PV, p‐AnV, and TOTOX value of the hulled and un‐hulled sesame oil samples (Alavi & Golmakani, 2017).

#### 2.2.18 Statistical analysis

The experiments were performed in triplicate, and the results were presented as mean value standard deviation. SPSS software version 22 was used for statistical analysis. The significance differences among means were set at *p <* .05 performing independent t‐test and repeated measures analysis of variance.

## 3 RESULTS AND DISCUSSION

### 3.1 Initial quality of hulled and un‐hulled sesame oils immediately after extracting the oil

#### 3.1.1 Fatty acid composition

Fatty acids profiles of the hulled and un‐hulled sesame oils are shown in Table 1. There were significant differences between hulled and un‐hulled sesame oils in terms of fatty acid composition. Linoleic acid and oleic acid were the main fatty acids in hulled and un‐hulled sesame oils, followed by palmitic acid and stearic acid. Our findings were consistent with the results of a study by Orsavova et al. (2015) in this regard. Previous studies have shown that the higher the amounts of oleic acid in vegetable oils, the higher the oxidative stability of the oil, since the oxidation rate of oleic acid is 10–40 times lower than that of linoleic acid, and the oxidation rate of linoleic acid is half of the oxidation rate of linolenic acid (Choe & Min, 2006; Yun & Surh, 2012). S*e* index is calculated based on the ratio of linoleic acid (as the index of PUFAs) and palmitic acid (as the index of unsaturated fatty acids) to evaluate the stability of an oil sample. This index indicates the amounts of PUFAs and, as a result, the tendency of the oil to oxidize spontaneously (Hemery et al., 2015).

**TABLE 1:** Fatty acid composition and se index of hulled and un‐hulled sesame oil samples (%).

Fatty acid	Un‐hulled sesame oil	Hulled sesame oil	Standard limit (CODEX)
Myristic acid	0.15 ± 0.15	0.02 ± 0.01	<0.1
Palmitic acid	10.48 ± 1.01	10.91 ± 1.71	7.9–12
Palmitoleic acid	0.20 ± 0.01	0.20 ± 0.01	<0.2
Heptadecanoic acid	ND^a^	0.06 ± 0.01	<0.2
Stearic acid	5.44 ± 0.58	5.78 ± 0.58	4.5–6.7
Oleic acid	40.41 ± 0.92	39.62 ± 1.49	34.4–45.5
Linoleic acid	42.43 ± 0.83	42.68 ± 2.46	36.9–47.9
Linolenic acid	0.22 ± 0.01	0.25 ± 0.02	0.2–1
Arachidic acid	0.56 ± 0.05	0.54 ± 0.10	0.3–0.7
Se index^a^	4.08 ± 0.35	3.97 ± 0.40	

Abbreviation: ND, not detected.

^a^ Mean ± standard deviation.

#### 3.1.2 Qualitative properties of virgin oil

Physicochemical properties of the hulled and un‐hulled sesame oils are shown in Table 2. PV value is one of the qualitative indicators evaluating the quality of edible oils during production, storage, and consumption, which expresses the amounts of hydroperoxides produced by lipid oxidation. Due to the instability of hydroperoxides over time and their conversion into secondary oxidation products, measuring the p‐AnV ensures oil quality control. Furthermore, the PV and p‐AnV can be used to calculate the TOTOX value, which indicates the total oxidation of oil samples. The AV is another indicator for the oil quality control, so the higher the AV of a sample, the higher the hydrolysis of that sample (Farhoosh & Hoseini‐Yazdi, 2014).

**TABLE 2:** Physicochemical properties of hulled and un‐hulled sesame oil samples.

Property	Un‐hulled sesame oil	Hulled sesame oil
AV (g KOH/kg)	3.88 ± 0.07^a^ ^*^	2.76 ± 0.01^b^
PV (meqO2/kg)	3.93 ± 0.09^a^	2.44 ± 0.62^a^
p‐AnV (mg/kg)	3.77 ± 0.43 ^a^	3.05 ± 0.07^a^
TOTOX value	11.77 ± 0.60^a^	7.05 ± 0.09^b^
Inhibition percent (%)	62.89 ± 0.13^a^	63.35 ± 1.73^a^
Smoke point (°C)	186.25 ± 1.25^a^	182.7 ± 2.7^a^
Oxalic acid (mg/kg)	4.09 ± 0.31^a^	2.83 ± 0.31^a^
Red color	11.22 ± 0.77^a^	13.88 ± 0.43^a^
TPC (mg/kg)	15.37 ± 1.12^a^	11.20 ± 2.30^a^
Carotenoid (mg/kg)	6.83 ± 0.36^a^	9.26 ± 1.01^a^
Relative density	0.915 ± 0.05^a^	0.915 ± 0.08^a^
Refractive index	1.4654 ± 0.15^a^	1.46 ± 0.06^a^

Abbreviations: AV, acid value; p‐AnV, para‐Anisidine value; PV, peroxide value; TOTOX, total oxidation; TPC, total phenolic content.

*Note*: Different small letters in a row means that the value differ significantly (*p <* .05).

* Mean ± standard deviation (*N* = 3).

The PV (3.93 ± 0.09), anisidine value (3.77 ± 0.43), and TOTOX value (11.77 ± 0.60) in the un‐hulled sesame oil were higher than the PV (2.44 ± 0.62), anisidine value (3.05 ± 0.07), and TOTOX value (7.05 ± 0.09) in the hulled sesame oil immediately after extracting the oil (Table 2), suggesting that the un‐hulled sesame oil had a higher tendency to oxidation. The higher oxidation rate in un‐hulled sesame oil may have been due to the presence of oil impurities which act like prooxidants and accelerate the oxidation rate. In addition, the presence of higher moisture content in oil samples contributes to acceleration of the oxidation. As for the hulled sesame seeds, heating was applied to dry the sesame seeds, which led to moisture content reduction in the hulled sesame seeds (from 2.51% to 1.34%).

The amount of oxalate in un‐hulled sesame oil was higher than that in hulled sesame oil. Makinde and Akinoso (2013) showed that the amount of antinutritional factors (e.g., phytic acid and oxalic acid) were higher in white sesame seeds, and the highest value was related to the hull of these seeds. Therefore, it can be argued that dehulling reduces the amount of antinutrient compounds such as oxalate.

Antioxidant activities of both oil samples were similar. Seemingly, the heat processing applied to dry the hulled sesame seeds led to production of new antioxidant compounds (e.g., Maillard reaction products), which improved the antioxidant activity of the hulled sesame oil (Hossini et al., 2019). In addition, the higher carotenoid contents in the hulled sesame oil improved its antioxidant activity (Alavi & Golmakani, 2017; Zhou et al., 2016).

The un‐hulled sesame oil was cloudy in comparison with the hulled sesame oil. Seemingly, dehulling the sesame seeds improved the appearance of the oil. Similarly, Nagendra Prasad et al. (2012) found that hulling the seeds improved the color of the oil. The color of hulled oil was clearer than that of un‐hulled oil. The results of sensory evaluation of the hulled and un‐hulled sesame oil samples as well as the refine sesame oil showed that the color of hulled sesame oil sample was brighter than those of un‐hulled sesame oil and refined sesame oil (Table 4). However, the refined sesame oil was tastier, more aromatic, and generally more acceptable than those of hulled and un‐hulled sesame oils.

#### 3.1.3 Aflatoxins

The levels of aflatoxins B1, B2, G1, and G2 as well as the total aflatoxins were measured; however, no aflatoxins were detected in the samples.

#### 3.1.4 Pesticide residue

The results of the residual pesticides in hulled and un‐hulled sesame oil samples are shown in Table 3. The obtained results indicated the presence of significant amounts (upper than standard limit: 50 μg/Kg) of triazophos pesticide in oil samples. The amount of this pesticide in the un‐hulled sesame oil (65 μg/Kg) was higher than that in the hulled sesame oil (52 μg/Kg). Triazophos is an organophosphorus pesticide that controls a wide range of insects. Small amounts of bifenthrin, pyriproxyfen cyhalothrin‐lambda I, cyhalothrin‐lambda II, cyhalothrin‐lambda total, and chlorpyrifos were also detected in both hulled and un‐hulled sesame oils.

**TABLE 3:** Residual pesticides (μg/kg) in hulled and un‐hulled sesame oil samples.

Pesticide	*Hulled sesame oil*	Un‐hulled sesame oil
Triazophos	65	52
Bifenthrin	<40	<40
Pyriproxyfen	<40	<40
Cyhalothrin‐lambda I	<14	<14
Cyhalothrin‐lambda II	<14	<14
Cyhalothrin‐lambda total	<14	<14
Chlorpyrifos	<14	<14

#### 3.1.5 Sensory evaluation of oil samples

Table 4 shows the results of sensory evaluation, including color, taste, smell, and overall acceptance of the refined, hulled, and un‐hulled sesame oils. Refined sesame oil had the highest score in terms of smell, taste, and overall acceptance indices, while the color index in hulled sesame oil had the highest score. Although there were no significant differences among the samples in terms of the color and taste indices, significant differences were observed among them regarding the overall acceptance and smell indices.

**TABLE 4:** Sensory Evaluation of Hulled and Un‐hulled Sesame Oil Samples.

Sesame oil	Color	Taste	Smell	Overall acceptance
Refined	23^a*^	31^a^	32^a^	34^b^
Hulled	19^a^	22^a^	15^b^	18^b^
Un‐hulled	29^a^	20^a^	23^ab^	25^ab^

* Different small letters in a column means the value differ significantly (*p <* .05).

#### 3.1.6 Oxidative stability of sesame oils during the storage period

**FIGURE 1:**
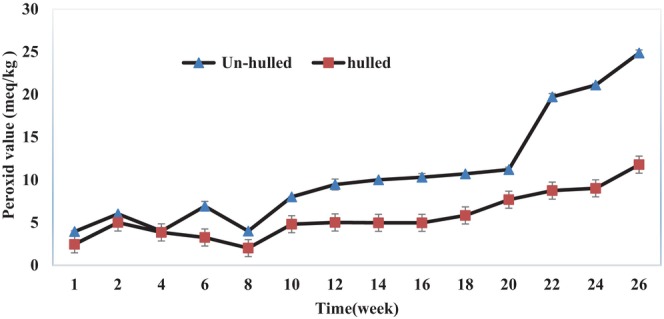
Peroxide value (PV) changes of hulled and un‐hulled sesame oils during the storage period.

**FIGURE 2:**
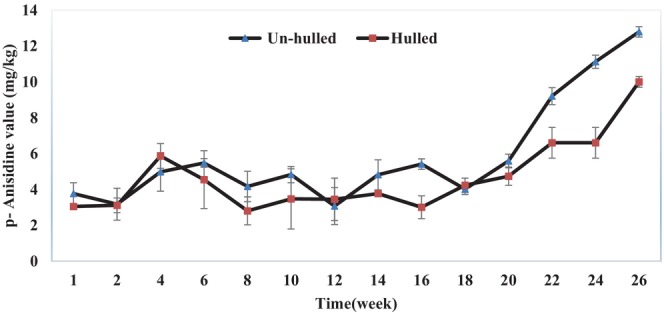
Para‐anisidine value (p‐AnV) of hulled and un‐hulled sesame oils during the storage period.

**FIGURE 3:**
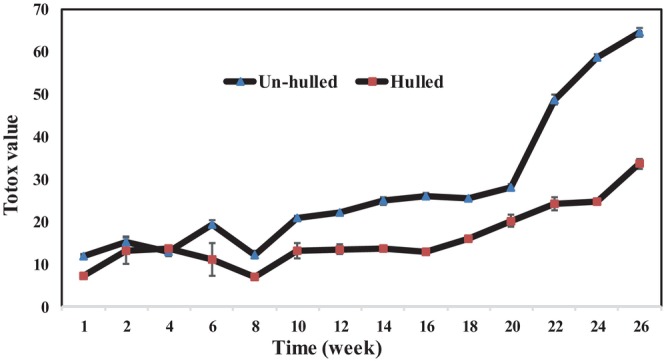
Total oxidation (TOTOX) value of hulled and un‐hulled sesame oils during the storage period.

Oxidative stability of the vegetable oils refers to their resistance against oxidative acceleration and to the quality loss resulting from this phenomenon. Oxidation of the PUFAs is one of the most important chemical reactions in edible oils, which decreases the quality (i.e., taste, smell, odor, and overall accessibility) of the vegetable oils (Farhoosh & Hoseini‐Yazdi, 2014). Our study results about the stability of hulled and un‐hulled sesame oils during the storage period are shown in Figures 1, 2, 3.

#### 3.1.7 PV of hulled and un‐hulled sesame oil samples

PVs of the hulled and un‐hulled sesame oil samples are presented in Figure 1. The PV of un‐hulled sesame oil was higher than that of hulled sesame oil at the beginning of the storage period. During the storage period, moreover, the PV of un‐hulled sesame oil changed more rapidly than that of hulled sesame oil. The PV of the un‐hulled oil samples reached 10 (meq/kg) after a 20‐week storage, and then rapidly increased to 20 (meq/kg) after 24 weeks. Statistical analysis of the PV changes in the hulled and un‐hulled sesame oil samples revealed a statistically significant difference in the given upward trend (*P ≤* 0.05).

#### 3.1.8 Anisidine value of hulled and un‐hulled sesame oil samples

Anisidine value of the oil samples during the storage period is presented in Figure 2. P‐AnV of the hulled and un‐hulled sesame oil samples increased during the storage period, and this increase was significantly higher in un‐hulled sesame oil than in hulled sesame oil.

#### 3.1.9 TOTOX value

The changes in TOTOX value are depicted in Figure 3. Since the PV and anisidine value were higher in un‐hulled sesame oil, the mentioned index was higher in un‐hulled sesame oil during the storage period than that in hulled sesame oil. In addition, the increasing trends of TOTOX index in the hulled and un‐hulled sesame oil samples were statistically significant during the storage period (*p* ≤ .05).

In general, the hulled sesame oil was more stable than the un‐hulled sesame oil, and its oxidation rates were less than those of the un‐hulled sesame oil. Seemingly, the heat processing applied to dry the hulled sesame seeds led to the production of new antioxidant compounds (e.g., Maillard reaction products), which improved the antioxidant activity of hulled sesame oil (Hossini et al., 2019). In addition, the higher carotenoid contents in the hulled sesame oil improved its antioxidant activity (Alavi & Golmakani, 2017). Akbulut (2008) reported that the mineral contents of sesame products such as Al, Ca, B, Cu, Fe, K, Mn, Mo, Na, Ni, P, and Zn were increased by increasing the hulls' content, but Mg content was decreased. Xia et al. (2021) and Niu et al. (2021) also demonstrated that the un‐hulled seed oil had higher content of heavy metals than the hulled seed oil so that these metals were able to serve as prooxidants and to decrease the oxidative stability of the oil. These results were inconsistent with the findings of studies by Nagendra Prasad et al. (2012) and Shahidi (2006) suggesting that the oxidative stability of un‐hulled sesame oil was higher than that of hulled sesame oil (Shahidi, 2006).

## 4 CONCLUSION

In sum, linoleic and oleic acids were the main fatty acids in the hulled and un‐hulled sesame oils. Aflatoxin was not detected in our oil samples; however, the un‐hulled oil samples contained higher amounts of pesticide residues and higher levels of oxalic acid than the hulled sesame oil samples.

Hulled sesame oil was more stable than un‐hulled sesame oil, and its oxidation rates were less than those of un‐hulled sesame oil. Seemingly, the heat processing of hulled sesame seeds resulted in the production of new antioxidant compounds (e.g., Maillard products) and the reduction of seed's moisture content, thereby improving the antioxidant activity and oxidative stability of the hulled sesame oil. In addition, the higher carotenoid contents in the hulled sesame oil improved its antioxidant activity.

## AUTHOR CONTRIBUTIONS

**Maryam Amini:** Data curation (equal); formal analysis (equal); investigation (equal); methodology (equal); software (equal); writing – original draft (equal). **Mohammad‐Taghi Golmakani:** Conceptualization (supporting); data curation (supporting); methodology (equal); validation (supporting); writing – review and editing (supporting). **Azam Abbasi:** Conceptualization (lead); data curation (lead); formal analysis (lead); funding acquisition (lead); investigation (lead); methodology (lead); writing – review and editing (lead). **Marzieh Nader:** Formal analysis (equal); investigation (equal).

## CONFLICT OF INTEREST STATEMENT

The authors declare that they have no conflicts of interest.

## PRACTICAL APPLICATION

Hulled sesame oil was more stable than un‐hulled sesame oil. Heat processing of the hulled sesame seeds led to the production of new antioxidant compounds and the reduction of moisture content of seed which improved the antioxidant activity and oxidative stability of the hulled sesame oil.

## Data Availability

The data supporting the findings of this study are available from the corresponding author upon reasonable request.
